# Validation of the comprehensive score for financial toxicity for Brazilian culture

**DOI:** 10.3332/ecancer.2020.1158

**Published:** 2020-12-18

**Authors:** Luciana de Alcantara Nogueira, Francisco José Koller, Larissa Marcondes, Maria de Fátima Mantovani, Sonia Silva Marcon, Paulo Ricardo Bittencourt Guimarães, Luciana Puchalski Kalinke

**Affiliations:** 1 Doctoral Students of the Nursing Graduate Program, Federal University of Paraná, Av Prof Lothário Meissner 632, Curitiba, PR 80210-170, Brazil; 2 Nursing Department, Federal University of Paraná, Av Prof Lothário Meissner 632, Curitiba, PR 80210-170, Paraná, Brazil; 3 Nursing Department, State University of Maringá, Av Colombo, 5790, Jd Universitário, Maringá, Paraná, 87020-900, Brazil; 4 Statistics Department, Federal University of Paraná, Av Cel Francisco H Santos 210, Curitiba, PR 81531-970, Brazil

**Keywords:** cancer, quality of life, validation studies, psychometrics, financial toxicity

## Abstract

Financial toxicity is a side effect of cancer treatment showing the financial burden experienced by cancer patients for funding their treatment. An instrument for its evaluation can contribute towards the creation of coping strategies. In Brazil, a developing country, cancer patients certainly feel the effects of this serious adverse event, however, the discussion on the theme and research concerning these issues is scarce and an instrument for evaluation can help in the promotion of coping strategies. Thus, the study objective was to: 1) translate and adapt the COmprehensive Score for financial Toxicity (COST) questionnaire to Brazilian culture and 2) evaluate the COST psychometric properties in Brazil. Thus, a methodological study was developed in two stages. In the first stage, the translation and cross-cultural adaptation were performed, and in the second stage, two groups of participants were recruited to evaluate the psychometric properties. In the first stage, 21 individuals participated, including translators and cancer patients, and in the second stage, 126 patients participated who were undergoing cancer treatment. For validation, exploratory factor analysis (EFA), confirmatory factor analysis (CFA) and Cronbach’s alpha coefficient were performed to verify internal consistency. For the first stage’s outcome, the pre-test Cronbach’s alpha was 0.83. The EFA and CFA carried out in the second stage of the study revealed that the COST Brazilian version measures a single construct with a value of—χ2/gl = 179.78, comparative fit index = 0.00, Parsimony goodness of fit index = 0.302, root mean square error of approximation = 1.196 and p-value of close fit = 0.000. When comparing the average of the COST score and the standard deviation of the two samples, the significance value was p = 0.001. Therefore, it is possible to confirm that the COST is a valid and reliable questionnaire to measure the financial toxicity of cancer patients in Brazil.

## Introduction

Financial toxicity is a side effect of cancer treatment, which describes the financial burden experienced by cancer patients, who may find it difficult to afford their treatment. Some authors [1] define it as the financial burden and the consequences experienced by cancer patients, even those who have private health insurance, who are unable to pay for treatment and often use their savings, change their lifestyle, acquire borrowed money to pay with costs and often declare bankruptcy.

Financial toxicity refers to objective financial burdens such as medical care, and the subjective suffering from monthly co-payments, leading to changes in the budget by changing family routines and habits [2]. It can cause disruptions both in the quality of life of the family and in the quality of life-related to the health (HRQoL) of patients. Other studies found the association between financial toxicity and worse HRQoL [3–6]. Its consequences impact on various dimensions of the patient’s and family’s life, sometimes passing through the monetary aspects and causing physical and psychological damages. Studies highlight changes in life habits, non-adherence or decreased use of the prescribed medication, discontinuity of treatment [7], indebtedness [8], increased anxiety [9], psychological symptoms [10] and decreased HRQoL [7].

In an attempt to quantify the experience of cancer patients related to financial difficulties during cancer treatment, the North American group Functional Assessment of Chronic Illness Therapy (FACIT) developed and validated [6] the COST questionnaire, with 11 items plus one considered a summary of all the questions. The answers are on a five-point Likert scale that measures the financial toxicity of the cancer patient, considered by some authors [11, 12] as a side effect of cancer treatment.

COST has already been translated and adapted for different cultures and applied in different health systems, in different countries. A study [13] in Canada, which has a public health system, used COST to define factors associated with financial toxicity in patients with lung cancer. In another study [8] in the United States, in which most populations have a private health system, it was used to investigate the financial impact of cancer among newly diagnosed individuals.

In Brazil, cancer treatment can be paid for in three different ways: 1) by the government, through the Unified Health System (governed by law 8080/90), which gives full rights to the diagnosis and treatment of the health of every Brazilian citizen; 2) by a private institution, by hiring a health insurance and 3) particular, condition in which the patient pays for the expenses.

Thus, Brazilian patients may or may not have financial toxicity, or even show different levels of this side effect. Studies on the topic in the country are incipient. Therefore, the use of COST can enable studies on this type of side effect of cancer treatment in the Brazilian population. Therefore, this study was carried out in two stages: 1) translating and adapting the COST for Brazilian culture and 2) evaluating the psychometric properties of the Brazilian version of COST, in individuals undergoing cancer treatment in two reference centres for cancer care in southern Brazil.

## Method

### Study design

This is a methodological study carried out in two stages. In stage 1, the translation and cross-cultural adaptation of the COST were carried out, and then, the content was validated. In stage 2, we established the psychometric properties of the questionnaire, including reliability and construct validity.

The Research Ethics Committee of Hospital de Clinicas, Federal University of Paraná (opinion number 2,687,637) approved the study, and all participants were informed about the objective and provided the signed informed consent form.

#### Stage 1—Translation and cross-cultural adaptation of COST

The translation and cross-cultural adaptation were carried out according to the measurement system of the FACIT group, which has the rights under COST. This measurement system had seven phases: The first phase is called advanced translation. The translation of COST into Brazilian Portuguese was carried out by two language professionals. In the second phase called reconciliation, a third language professional whose native language was Brazilian Portuguese and fluent in English evaluated the two previous versions and consolidated them into a single version. In the third phase called back translation, the reconciled version was translated back into English by a fourth bilingual specialist who lives in the United States, and who had not yet participated in any previous phases.

In the next phase called quality control, all translations were sent to the FACIT group for review and harmonisation. In the fifth phase called independent review, two health professionals and a language professional analysed the advanced translations, reconciliation, back translation and comments sent by the FACIT group. The reviewers discussed the comments and provided clarifications on each one. In the sixth phase, called the finalization process, the questionnaire containing all the phases carried out was again sent to the FACIT group, for completion of the reviews by the language coordinator. The developers formatted COST and approved the process. The seventh phase called pre-test was performed with 15 patients undergoing cancer treatment. These patients were having chemotherapy treatment at the time of data collection.

#### Stage 2—Testing the psychometric properties of COST

In stage 2, two groups examined the psychometric properties of the COST: cancer patients undergoing treatment in a haematology and oncology outpatient clinic of a public hospital at the Federal University of Paraná (public institution), and cancer patients who performed the treatment in a private institution for diagnosis, treatment, and monitoring of cancer patients who have private health insurance or who pay for the treatment (a private institution).

The construct’s validity was tested by examining the factor structure through exploratory factor analysis (EFA) and confirmatory factor analysis (CFA) in both samples and the total sample. Cronbach’s alpha examined reliability.

### Questionnaires

According to the research stages, there were different questionnaires used. In stage 1, the form sent by the FACIT group was used to carry out the six phases (advanced translation, reconciliation, back translation, quality control, independent review, finalisation process). In the pre-test, the COST translated into Brazilian Portuguese was used, two extra questionnaires, which aimed to assess the difficulties of understanding the questionnaire and the interpretations of patients in all items of the questionnaire, all sent by the FACIT group, a questionnaire demographic and clinical socio that gathered information such as sex, age, number of children, profession, occupation, income and clinical data such as diagnosis, presence of comorbidities, use of medication described by the researchers.

In stage 2, the COST and the socio-demographic and clinical questionnaire were used.

The original COST version 2 is a measure with 12 items on a single scale. The North American validation version 1 of the questionnaire was published with 11 items. However, version 2 included an additional item considered a summary, but without punctuation, according to the Scoring Guidelines of the FACIT group sent to the researchers at the time of acceptance in translating the questionnaire. The procedure of not using this item in the score was also performed by the Italian validation when the authors state that ‘following the scoring procedure proposed by the authors, item 12 has to be excluded’ [14]. Items 2, 3, 4, 5, 8, 9 and 10 are reversed. COST’s answers range from ‘not at all’ to ‘very much’ and the score ranges from 0 to 44, the higher the financial well-being, that is, the lower the financial toxicity. The questionnaire answers are based on the 7 days before the administration of the test, and it is a self-report questionnaire that can be completed by the patient or with the assistance of the examiner.

### Participants and data collection

In the translation and cross-cultural adaptation stage, four translators and two health professionals carried out the translations, reconciliation, back-translation and analysis by specialists. In the pre-test, 15 patients undergoing cancer treatment comprised the sample. In the validation stage of the psychometric properties, the other 126 patients undergoing cancer treatment from two cancer treatment centres in a capital city in southern Brazil participated in the study.

The stage of translation and cross-cultural adaptation was carried out between April and July 2018. Data from the second stage, assessment of psychometric properties, were collected between September 2018 and January 2020.

The sample calculation considered the number of items in the COST questionnaire and was based on the reference by Pasquali [11], who suggests that the number of participants as a sample should be 5 to 10 times the number of items. Considering that COST has 12 items, a sample size of 126 participants for the second stage was considered adequate. Participants were having chemotherapy at the time of data collection. The sample of both translators and patients for the pre-test and validation was non-probabilistic.

The inclusion criteria were: being over 18 years old, diagnosed with cancer for 6 months or more; undergoing treatment and be able to read and speak Portuguese. We did not include patients with cognitive impairment and/or speech disorders who were undergoing chemotherapy for a condition other than cancer.

#### Data analysis

COST answers were typed in Microsoft Office Excel, version 2010 at two different times, for the conference. The data from the first stage of the study (pre-test) were analysed in two different ways: qualitative analysis with 10 patients using the form sent by the FACIT group to assess the degree of offense and relevance of each of the items in the questionnaire; and quantitative analysis with the addition of five patients, totaling 15 to perform Cronbach’s alpha.

In the second stage, we performed the Spearman’s correlation test to assess the correlation between the items, and the EFA and CFA, using the method of estimating unweighted least squares. We observed the following criteria to check the suitability of the sample: sample size *n* > 100; correlation matrix coefficients with a value above 0.3 (about half of the coefficients); Kaiser-Meyer-Olkin test (KMO) with a value above 0.6; Bartlett’s sphericity test. CFA was performed using the SPSS Amos program, using the maximum likelihood estimation method.

We obtained Cronbach’s alpha coefficient to assess the internal consistency of both stages, in which results greater than 0.70 are acceptable values [15]. Student’s *t* and Shapiro–Wilk tests compared the two groups regarding the COST score and standard deviation (SD).

## Results

### First stage

Twelve of the 15 patients who participated in the pre-test were female, seven did not have comorbidities, 12 declared not to practice physical activity and 10 had an income between US$ 194.59 and US$ 583.79 monthly. There were no difficulties in understanding the items. In the evaluation of internal consistency, Cronbach’s alpha was 0.83. This stage involved the participation of specialists and the FACIT group, ensuring the content validity to COST.

*General characteristics*In the stage of validation of the psychometric properties, 126 patients undergoing cancer treatment comprised the sample. Forty-three of them were recruited from a private institution and 83 from the public institution.

According to the socio-demographic data, the total sample consisted of 59.52% (*n* = 75) women, 60.32% (*n* = 76) were married or declare a common law-marriage; with a mean age of 56 years old, in which 53.17% (*n* = 67) of the patients were in the age group between 31 and 59 years old; the main occupation found was retired 35.48% (*n* = 44), the family monthly income was US$ 194.59 to US$ 583.79 representing 43.20% (*n* = 54). Regarding clinical data, 47.58% (*n* = 59) did not have any comorbidity; however, 90.65% (*n* = 118) used frequent medication (Table 1). As for the type of tumour, 62.70% (*n* = 79), of the patients treated solid tumours

### Construct validation

#### Spearman’s correlation test

When performing the Spearman correlation test (Chart 1) in all the items in the questionnaire from both institutions (*n* = 126), we observed stronger correlations between the items 7 and 11 (*ρ* = 0.595). Regarding the *p*-value, there is a significant correlation in 86 of the 110 combinations, representing a significant correlation in 78.18%. The item 10 had a significant correlation with all the others, and the items 2, 3 and 8, of the 10 possible correlations, nine of them were significant. This shows that the items of the instrument have a good correlation with each other.

#### EFA, confirmatory factor analysis and Cronbach’s alpha coefficient of the total sample

When performing the KMO and Bartlett tests, we verified that the relationship between the 11 items is excellent, and it is possible to perform factor analysis. Two factors were defined using the EFA considering the eigenvalues, values above 1 and the variance explained by the model adjusted with two factors was 51.84%, by the non-rotated solution (Table 2).

When verifying the factorial load (Chart 2), through the EFA, we observed that the adjustment of a factorial model (two factors) indicates that the all items, except for item 1, have a good correlation between them, or that is, a model with two factors (two domains) would bring few advantages. When observing the item-total correlation, we saw that item 1 is the one with the lowest correlation with the other items in the questionnaire, and that Cronbach’s alpha increases when this item is removed. However, this item must remain in the questionnaire for the value of the factor load and a single domain is the one that best represents the COST.

When the CFA was performed for the results regarding the psychometric properties obtained in the EFA, the CFA revealed unsatisfactory results for all the adjustment indexes presented, which puts in doubt the use of a model with two factors/constructs. This result reinforces that the original COST structure is adequate for the sample of this research.

The CFA result using the AMOS 24/SPSS program, using the maximum likelihood estimation method (Table 3) showed that the value of—*χ*^2^/*gl* was less than 2, considered good. The comparative fit index (CFI) that measures the relative improvement in the adjustment of the model in a standard model obtained a value of 0.00 without the possibility of adjustment. The PGFI regarding the degree of parsimony of the tested model showed a lower value than the indicators of a good fit. The RMSEA is a test that tests the fit close to the model by comparing the model under test and a saturated model with the same data set obtained a value greater than that considered good for adjustment. Pclose, which tests the proximity of the adjustment with an index lower than 0.5, that is, without the possibility of adjustment, the ideal is that this index is higher than 0.5. This analysis corroborates with the EFA and strengthens the understanding that the organisation of the questionnaire is appropriate.

The Cronbach’s alpha coefficient found was 0.815, and we did not observe significant increase if the items were excluded from the questionnaire.

#### Comparison of the COST score between samples using the Student´s t-test for independent means proven by the Shapiro–Wilk test

When comparing the average of the COST score and the SD of the two samples, we observed significance with a value of *p* = 0.001. The SD of the sample of the private institution was 9.48, indicating greater variability of answers for the public institution that obtained a value of 6.57 (Table 4).

## Discussion

This is the study that translated, cross-culturally adapted and analysed the psychometric properties of the COST questionnaire for Brazilian culture.

COST is a questionnaire that was developed based on the concept of financial toxicity, to measure the side effect of cancer treatment. It has been widely used by American authors and is expanding to other cultures after the process of translation and cross-cultural adaptation, such as Japan [16]. Until July 2020, it was validated in the country of origin, USA [6] and Italy [14]. From this study, it was also validated in Brazil.

In the first stage of the study, we adopted the methodology of translation and cross-cultural adaptation of the FACIT [17] group, and we did not identify difficulties in any of the phases. The language coordinator of the FACIT group participated in two different moments: after the back-translation, when the researchers justified the use of some terms and, subsequently, when they investigated the claims for the use of the expressions. After the verification, the COST Portuguese version of Brazil was released for the pre-test, with the same 12 items of the original version, 11 items and one was considered a summary, as occurred in the Italian validation [14] and the Japanese translation and adaptation [16].

The pre-test was performed with 15 patients undergoing cancer treatment, and the Cronbach’s alpha coefficient was 0.83, considered excellent [18]. We found a similar result in the study in Japan [16] with a Cronbach’s alpha of 0.87 in the COST pre-test with the Japanese population.

Regarding the second stage of the study, 70.16% of the patients in the sample had some comorbidity with a predominance of systemic arterial hypertension (SAH), a similar situation than in the sample from the Italian validation [14]. In the survey [8] that investigated the financial impact of cancer in newly diagnosed individuals in the USA, SAH was also observed in their sample as the main health problem.

A study [19] to review the association between SAH and cancer found that it is a more common comorbidity in cancer patients. The authors concluded that hypertension is a common adverse event of anticancer treatments, and care must be taken not to underestimate its existence because well-controlled blood pressure values reduce the risk of cardiotoxicity and ineffectiveness of chemotherapies.

The Spearman test showed the correlation between the items in the questionnaire and the statistical significance between them. The other COST validation studies used Pearson’s correlation test, and it was difficult to compare with this study.

To perform the EFA, KMO and Bartlett tests on the total sample, they indicated that the relationship between the 11 items is very good, corroborating with the results of the Italian COST validation, which obtained a KMO of 0.82 and Bartlett of 486.2. The variance explained by the adjusted model with two factors is 51.636 for the non-rotated solution, differing from the Italian version, which was 63%. The KMO test evaluated the adequacy of the factor analysis and Bartlett’s sphericity test checks the hypothesis that the variables are not correlated in the population.

The factorial load found considering the results of the total sample, followed the outcomes of the North American [6] and Italian [14] validation, which pointed out that the COST can be considered a one-dimensional questionnaire, that is, that measures a single construct. Thus, there is no advantage in adding new domains to the questionnaire, that is, the original COST structure does not need to be changed. This result was confirmed by the CFA. The factor load indicates the correlation between the variable and the corresponding factor, the greater the factor load, the greater the correlation with a given factor, considering the range from 0 to 1, and the closer to 1, the greater its representation, regardless the signal. High factor load indicates convergence to a common point.

The internal consistency with the total sample was measured by Cronbach’s alpha coefficient and the value found was 0.815 indicating an excellent result [18]. This result is similar to the Italian version, which was 83%, and the North American version, which was 92%. Both surveys that validated the COST did not address populations with different economic characteristics as in this work; however, the reliability of the questionnaire proved to be excellent emphasising how reliable it is.

When observing the COST score of the two samples, we verified a higher score in the private institution, showing that there is greater financial well-being among those who have a private health plan or pay for treatment, that is, less financial toxicity. However, the maximum score is 44, revealing that there are different degrees of financial toxicity and that it is extremely relevant to identify patients who show signs of financial concern in different scenarios. The SD was higher in the sample of the private institution because the answers regarding income had a greater variability in this institution. That is, patients in the private institution filled in all the categories of this variable, while those in the public institution concentrated in the three lowest categories This situation was already presented in 2009 when the concept of financial toxicity emerged [20] and was reinforced by researchers in the elaboration of the questionnaire [21].

The Japanese study [16] divided the COST score into four grades: grade 0: score above 26, grade 1 (means mild impact): score between 14 and 25, grade 2 (moderate impact): score between 1 and 13, grade 3 (high impact): score 0. According to this study, grades 2 and 3 indicated positive financial toxicity. For this classification, both samples of this research had financial toxicity with mild impact. The North American validation study [6] verified the COST score separately between the samples and obtained the following results: at The University of Chicago the average score was 22.34 and in the North Shore University Health System sample was 21.60.

Some authors [22] studied samples with different ages to verify the COST score in samples with different characteristics and observed an average score of 24.84 among individuals aged 15 to 25 years old, and 18.22 among those in the age range 26 to 39 years old. We can conclude with the study that younger people have greater financial toxicity in that sample.

Regarding the different degrees of financial toxicity, the question of whether or not health insurance can significantly influence financial suffering. Patients with private insurance may suffer as much or even more than those who depend exclusively on the public system, because, depending on the therapy adopted, the costs with drugs and tests are high and the amounts are often paid by the contractor.

In Brazil, a little more than 47 million people had private health insurance [23] out of a population of 210,147,125, which represents 22% of the country’s population. They are entitled to the Unified Health System (SUS) like any Brazilian citizen, but they choose to pay health insurance to protect from excessive expenses with medical assistance [24]. Also, agility, choice of professional, service location and type of hospitality in case of hospitalisation can collaborate in the decision to hire a private health insurance.

Almost 50% have a co-participation plan, that is, in addition to the monthly fee, they pay a percentage (ranging up to a maximum of 40%) for each service performed such as a consultation, examination, hospitalisation or outpatient treatment. This type of plan protects the patient from exorbitant payments, but still burdens the patient who has increased costs. Some authors [9] reinforced that health systems with cost-sharing can make the inefficient use of resources, increasing the risk of adverse events.

In this study, we found that both samples showed financial toxicity. However, it had less intensity among those who had private health insurance or who paid for the treatment, revealing that together with this sample, those with greater purchasing power have less suffering in the cost of cancer therapy.

We believe that the difference in the financial toxicity experienced by the two groups is not exclusively related to family income. This is because most patients treated by the private institution with less financial suffering had a health insurance and did not make full payment for calls. Although the sample of the private institution has higher income, it was inappropriate to associate the COST score only with the participants’ income. When we used the classification carried out by the Japanese article [16], both samples had a moderate financial toxicity score, that is, they suffer a similar impact.

This study reinforces that the COST Brazilian version and the original questionnaire address issues related to the presence of financial difficulties after the onset of cancer. This situation was verified in the statement of the questionnaire, when it stresses that the answers of the participants must be from the last 7 days, and in items 3 and 10 mentioning the disease. Such opportunities emphasise that the questionnaire measures the financial toxicity construct.

### Limitation

The main limitation is related to not using other questionnaires, such as the evaluation of the health-related quality of life (HRQoL), which would enable us to verify the relationship between financial toxicity and HRQoL. We suggest further research on similar samples with the application of COST and questionnaires for the evaluation of HRQoL to verify this association with the Brazilian population and research that measure financial toxicity with the application of the translated COST and adapted to Brazilian culture.

## Conclusion

This study was aimed at carrying out the translation, cross-cultural adaptation and validation of the COST questionnaire for Brazilian culture. The COST Brazilian version follows the original structure and the Italian version, consisting of 11 items plus one considered a summary, proved to be adequate.

Content and construct validity were performed. Spearman’s correlation test revealed significance between items, and the internal consistency of the questionnaire showed an excellent result in the sample studied. We can consider the Brazilian version of COST as a valid and reliable measure for measuring financial toxicity among cancer patients.

## Conflicts of interest

The author(s) declare that they have no conflicts of interest.

## Funding statement

This study received financial assistance through a productivity scholarship contemplated in the notice of the Araucária Foundation with the number 2017-CP15.

## Figures and Tables

**Table 1.** Socio-demographic and clinical characteristics of the sample in the second stage of the study (*n* = 126).

	Private institution		Public institution		Sample total	
	*n*	%	*n*	%	*n*	%
**Gender**						
Male	18	41.86	33	39.76	51	40.48
Female	25	58.14	50	60.24	75	59.52
**Age**						
18 to 30 years old	0	0	4	4.82	4	3.17
31 to 59 years old	23	53.49	44	53.01	67	53.17
Over 60 years old	20	46.51	35	42.17	55	43.65
**Marital status**						
Married	27	62.79	44	53.01	71	56.35
Single	5	11.63	14	16.87	19	15.08
Separate	3	6.98	12	14.46	15	11.90
Common law-marriage	2	4.65	3	3.61	5	3.97
Widowed	6	13.95	10	12.05	16	12.70
**Occupation**						
Formal employment	11	25.58	16	19.75	27	21.77
Self-employed	12	27.91	13	16.05	25	20.16
Unemployed	0	0	10	12.35	10	8.06
Housewife	5	11.63	13	16.05	18	14.52
Retired	15	34.88	29	35.80	44	35.48
**Family monthly income**						
Without income	0	0	5	6.02	5	4
Up to US$^a^ 194.59	1	2.38	16	19.28	17	13.60
US$ 194.59 to US$ 583.79	3	7.14	51	61.45	54	43.20
US$ 778.39 to US$ 1,946.00	23	54.76	11	13.25	34	27.20
US$ 1,946.00 to US$ 3,892.00	8	19.05	0	0	8	6.40
More than US$ 3,892.00	7	16.67	0	0	7	5.60
**Comorbidities**						
No	22	51.16	37	45.68	59	47.58
Hypertension	16	37.21	24	29.63	40	32.26
Diabetes	5	11.63	11	13.58	16	12.90
Other	9	20.93	22	27.16	31	25
**Medicines in use**						
No	9	20.93	15	18.07	24	19.05
Pain relievers	6	13.95	20	24.10	26	20.63
Anti-inflammatory	0	0	9	10.84	9	7.14
Antibiotic	4	9.30	3	3.61	7	5.56
Other	26	60.47	50	60.24	76	60.32

a US$—United States dollar

**Table 2.** Relationship between COST items by KMO and Bartlett tests, considering the total sample (*N* = 126).

Test	Statistic	*p*^a^
KMO	0.812	
Bartlett	404.176	0.000

KMO, Kaiser–Meyer–Olkin

a *p,* significance level

**Table 3.** Description of the CFA values (*n* = 126).

Indexes	*χ*^2^*/gl*	CFI	PGFI	RMSEA	*p_close_*
Adjusted	179.78	0.00	0.302	1.196	0.000

*χ*^2^*/gl*, Chi-square/freedom level; CFI, Comparative fit index; PGFI, Parsimony goodness of fit index; RMSEA, Root mean square error of approximation; *p*_close_, *p*-value of close fit

**Table 4.** Comparison of the COST score, SD and statistical significance of the two samples (*n* = 126).

	Mean					SD	
	Private Inst.	Public Inst.	*t*	Gl	*p*	Private Inst.	Public Inst.
COST score	24.02	16.33	4.76	63.47	0.001	9.48	6.57

SD, Standard deviation; Inst., Institution; *t*, The difference in the variation in data; Gl, Freedom level; *p*, Significance

**Chart 1.** Spearman correlation of COST translated and adapted for Brazilian culture considering the total sample (*n* = 126).

	Spearman *p*-value^a^										
	**Item 1**	**Item 2**	**Item 3**	**Item 4**	**Item 5**	**Item 6**	**Item 7**	**Item 8**	**Item 9**	**Item 10**	**Item 11**
Item 1		−0.049 0.583	−0.097 0.277	0.148 0.099	−0.126 0.160	0.377 0.000	0.421 0.000	−0.108 0.230	−0.027 0.763	−0.235 0.008	0.315 0.000
Item 2	−0.049 0.583		0.350 0.000	0.294 0.001	0.299 0.001	−0.305 0.001	−0.195 0.029	0.394 0.000	0.182 0.042	0.336 0.000	−0.205 0.021
Item 3	−0.097 0.277	0.350 0.000		0.359 0.000	0.452 0.000	−0.278 0.002	−0.246 0.006	0.497 0.000	0.355 0.000	0.506 0.000	−0.223 0.012
Item 4	0.148 0.099	0.294 0.001	0.359 0.000		0.304 0.001	−0.015 0.864	−0.126 0.159	0.326 0.000	0.293 0.001	0.248 0.005	−0.061 0.501
Item 5	−0.126 0.160	0.299 0.001	0.452 0.000	0.304 0.001		−0.305 0.001	−0.304 0.001	0.430 0.000	0.313 0.000	0.579 0.000	−0.255 0.004
Item 6	0.377 0.000	−0.305 0.001	−0.278 0.002	−0.015 .864	−0.305 0.001		0.462 0.000	−0.204 0.022	−0.160 0.074	−0.270 0.002	0.436 0.000
Item 7	0.421 0.000	−0.195 0.029	−0.246 0.006	−0.126 0.159	−0.304 0.001	0.462 0.000		-0.328 0.000	-0.097 0.278	-0.230 0.010	0.595 0.000
Item 8	−0.108 0.230	0.394 0.000	0.497 0.000	0.326 0.000	0.430 0.000	−0.204 0.022	−0.328 0.000		0.400 0.000	0.503 .000	−0.233 0.009
Item 9	−0.027 0.763	0.182 0.042	0.355 0.000	0.293 0.001	0.313 0.000	−0.160 0.074	−0.097 0.278	0.400 0.000		0.237 0.007	−0.044 0.629
Item 10	−0.235 0.008	0.336 0.000	0.506 0.000	0.248 0.005	0.579 0.000	−0.270 0.002	−0.230 0.010	0.503 0.000	0.237 0.007		−0.203 0.023
Item 11	0.315 0.000	−0.205 0.021	−0.223 0.012	−0.061 0.501	−0.255 0.004	0.436 0.000	0.595 0.000	−0.233 0.009	−0.044 0.629	−0.203 0.023	

a *p-*value, significance level < 0.05

**Chart 2.** Factorial load of the 11 COST items considering the total sample (*n* = 126).

Items	Factorial load 1	Factorial load 2
1. I know that I have enough financial resources in savings, retirement or assets to cover the costs of my treatment	−0.388	0.464^a^
2. The amounts I had to pay to cover medical expenses exceeded what I imagined paying	0.497^a^	0.144
3. I am concerned about the financial problems I will have in the future as a result of my illness or treatment	0.657^a^	0.244
4. I feel like I have no choice in the amount I spend on healthcare	0.390^a^	0.389
5. I feel frustrated because I cannot work or contribute as much as I used to	0.680^a^	0.159
6. I am satisfied with my current financial situation	−0.542^a^	0.403
7. I can manage my monthly expenses	−0.579^a^	0.463
8. I feel financially stressed	0.645^a^	0.264
9. I am concerned with keeping my job, as well as my income and also with my household chores	0.407^a^	0.229
10. My cancer or treatment reduced my satisfaction with my current financial situation	0.665^a^	0.206
11. I feel I have control over my financial situation	−0.508^a^	0.443
Explained variance	3.913	1.789
Proportion	0.356	0.163

a The item belongs to the factor
